# Supplementary material for: BUBR1 as a Prognostic Biomarker in Canine Oral Squamous Cell Carcinoma
Source: Animals (Basel). 2022 Nov 9;12(22):3082. doi: 10.3390/ani12223082 (PMC9687056; doi:10.3390/ani12223082)
Supplement: Supplementary file 1 [file animals-12-03082-s001.zip › animals-1933652-supplementary.pdf]

animals MDPI

SUPLEMMENTAL MATERIAL

Figure S1

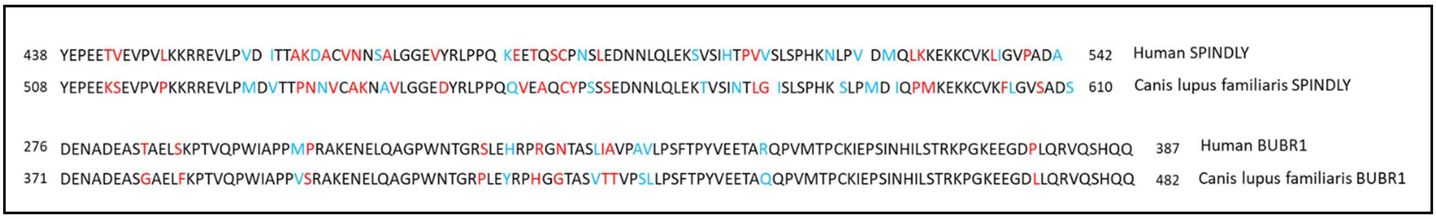

Figure S1. Aminoacids (aa) alignment of the binding site of the SPINDLY and BUBR1 antibodies in the Human SPINDLY and BUBR1 proteins sequences with the possible binding site sequence of Canis lupus familiaris. Red represents no similar aa and blue similar aa. The lack of manufacture information regarding BUB3 peptide sequence recognized by the antibody didn't allow us to show the aa alignment for this antibody. However, because human and dog Bub3 proteins share 100% amino acid identity, the putative epitopes recognized by Bub3 antibody are expected to be the same.

Suplemmental Table S1.

Table S1. Identity and similarity in percentage for the Human and Canis lupus familiaris protein sequences. For the alignment with the known antibody binding sequences the local alignment (Smith-Waterman) was performed. For the remaining alignments the global alignment (Needleman-Wunsch) was performed.

	Identity (%)	Similarity (%)
SPINDLY	70.37	77.07
SPINDLY (antibody sequence)	64.76	78.10
BUBR1	80.40	84.20
BUBR1 (antibody sequence)	86.61	91.96
BUB3 isoform 1	100	100
BUB3 isoform 2	99.39	99.39
BUB3 isoform 3	83.84	83.84

Suplemmental Table S2. Clinicopathological characteristics and BubR1 expression comparison.

		BubR1 Extent Score			BubR1 Intensity Score		
Variables		≤49%	≥50%	*P*-Value	(0/Weak/Moderate)	(Strong)	*P*-Value
**Gender**	Female	13	15	0.784	14	14	1.000
	Male	16	16		16	16	
**Age (years)**	<7	17	13	0.200	17	13	0.306
	≥7	12	18		13	17	
**Breed**	Small	8	2	0.030 **	5	5	0.501
	Medium	3	2		5	0	
	Large	2	10		6	6	
	Undetermined	13	13		13	13	

**Tumour Location**	Mouth (NOS)	6	7	0.664	6	7	0.442
	Gingiva	12	8		13	7	
	Tongue	5	7		5	7	
	Oropharynx	4	4		3	5	
	Palate	2	5		3	4	
**Histological type**	Papillary SCC	8	2	0.029	6	4	0.482
	Conventional SCC	21	29		24	26	
**Bone Invasion**	Yes	8	2	0.029	8	2	0.039
	No	21	29		22	28	
**Vascular Invasion**	Yes	1	3	0.430	1	3	0.305
	No	28	28		29	27	
**Histological grade (Anneroth)**	Well differentiated	10	9	0.653	7	12	0.169
	Moderate differentiated	19	22		23	18	
	Poor differentiated	-	-		-	-	
**Histological grade (Bryne)**	Well differentiated	14	11	0.283	12	13	0.959
	Moderate differentiated	15	18		18	15	
	Poor differentiated	0	2		0	2	
**Mitosis**	0-1/hpf	7	10	0.675	7	10	0.237
	2-3/hpf	12	9		10	11	
	4-5/hpf	8	8		9	7	
	>5/hpf	2	4		4	2	
**Lymphoplasmacytic infiltration**	Weak	8	10	0.765	10	8	0.509
	Moderate	13	11		12	12	
	Marked	8	10		8	10	
**Necrosis**	Yes	12	9	0.320	12	9	0.421
	No	17	22		18	21	
**Pattern of invasion ***	I	13	7	0.142	8	12	0.876
	II	9	13		14	8	
	III	7	8		8	7	
	IV	0	3		0	3	
**Stage of invasion ***	I	0	0	0.029 **	17	22	0.159
	II	23	16			8	
	III	5	15		12	0	
	IV	1	0		1		
**Treatment ***	Surgery	7	4	0.289	6	5	0.381
	Chemotherapy	1	3		2	2	
	Palliative treatment/support	14	21		14	21	
**Tumour stage**	I + II	12	7	0.034	10	9	0.611
	III + IV	10	21		14	17	

* Pattern of invasion also evaluated using the categorization of I + II vs III + IV (P=0.342 and P=0.576 for extent and intensity scores respectively) and I + II + III vs IV (P=0.070 and P=0.277); stage of invasion also evaluated using the categorization of I + II vs III + IV (P=0.026 and P=0.180); and treatment using the categorization of treatment in Surgery/Chemotherapy vs. Palliative treatment/support (P=0.389 and P=0.389). ** Pairwise multiple comparisons with bonferroni adjustment showed differences between small vs large breed (P=0.020), stage of invasion between stages II vs III (P=0.043); IV vs II (P=0.003) and IV vs III (P=0.003).

Suplemmental Table S3. Clinicopathological characteristics and Bub3 expression comparison

		Bub3 Extent Score			Bub3 Intensity Score		
**Variables**		**≤74%**	**≥75%**	***P*-Value**	**(0/Weak/Moderate)**	**(Strong)**	***P*-Value**
**Gender**	Female	10	17	0.557	21	6	0.128
	Male	13	16		17	12	
**Age (years)**	<7	9	19	0.178	18	10	0.571
	≥7	14	14		20	8	
**Breed**	Small	4	3	0.622	7	0	0.259
	Medium	2	3		3	2	
	Large	3	8		8	3	
	Undetermined	12	14		16	10	
**Tumour Location**	Mouth (NOS)	5	5	0.211	7	3	0.825
	Gingiva	5	14		14	5	
	Tongue	6	6		8	4	
	Oropharynx	2	6		4	4	
	Palate	5	2		5	2	
**Histological type**	Papillary SCC	3	5	0.863	7	1	0.203
	Conventional SCC	20	28		31	17	
**Bone Invasion**	Yes	2	7	0.214	8	1	0.144
	No	21	26		30	17	
**Vascular Invasion**	Yes	2	2	0.771	4	0	0.270
	No	21	31		34	18	
**Histological grade (Anneroth)**	Well differentiated	5	13	0.168	13	5	0.633
	Moderate differentiated	18	20		25	13	
	Poor differentiated	-	-		-	-	
**Histological grade (Bryne)**	Well differentiated	11	13	0.770	18	6	0.314
	Moderate differentiated	11	19		18	12	
	Poor differentiated	1	1		2	0	
**Mitosis**	0-1/hpf	5	11	0.141	12	4	0.742
	2-3/hpf	6	12		12	6	
	4-5/hpf	7	9		11	5	
	>5/hpf	5	1		3	3	
**Lymphoplasmacytic infiltration**	Weak	7	9	0.960	10	6	0.863
	Moderate	9	14		16	7	
	Marked	7	10		12	5	
**Necrosis**	Yes	9	10	0.496	14	5	0.507
	No	14	23		24	13	
**Pattern of invasion ***	I	6	11	0.288	13	4	0.219
	II	12	9		11	10	
	III	4	11		11	4	
	IV	1	2		3	0	
**Stage of invasion ***	I	0	0	0.461	0	0	0.309
	II	13	22		25	10	
	III	10	10		13	7	
	IV	0	1		0	1	
**Treatment ***	Surgery	4	6	0.596	7	3	0.783
	Chemotherapy	2	2		2	2	
	Palliative treatment/support	10	25		22	13	

**Tumour stage**	I + II	7	9	0.442	14	2	0.065
	III + IV	10	21		19	12	

* Pattern of invasion also evaluated using the categorization of I + II vs III + IV (P=0.168 and P=0.278 for extent and intensity scores respectively) and I + II + III vs IV (P=0.565 and P=0.367); stage of invasion also evaluated using the categorization of I + II vs III + IV (P=0.445 and 0.464); and treatment using the categorization of treatment in Surgery/ Chemotherapy vs Palliative treatment/support (P=0.340 and P=0.926).

Suplemmental Table S4. Clinicopathological characteristics and Spindly expression comparison

		**Spindly Extent Score**			**Spindly Intensity Score**		
**Variables**		**≤49%**	**≥50%**	***P*-Value**	**(0/Weak/Moderate)**	**(Strong)**	***P*-Value**
**Gender**	Female	10	16	0.139	23	3	0.888
	Male	17	12		26	3	
**Age (years)**	<7	14	15	0.899	26	3	0.888
	≥7	13	13		23	3	
**Breed**	Small	6	3	0.330	8	1	0.748
	Medium	3	2		5	0	
	Large	7	4		10	1	
	Undetermined	9	15		20	4	
**Tumour Location**	Mouth (NOS)	5	6	0.297	10	1	0.360
	Gingiva	10	10		19	1	
	Tongue	7	2		7	2	
	Oropharynx	2	6		6	2	
	Palate	3	4		7	0	
**Histological type**	Papillary SCC	3	5	0.550	8	0	0.582
	Conventional SCC	24	23		41	6	
**Bone Invasion**	Yes	5	5	0.950	10	0	0.225
	No	22	23		39	6	
**Vascular Invasion**	Yes	1	3	0.405	3	1	0.628
	No	26	25		46	5	
**Histological grade (Anneroth)**	Well differentiated	7	12	0.191	17	2	0.948
	Moderate differentiated	20	16		32	4	
	Poor differentiated	-	-		-	-	
**Histological grade (Bryne)**	Well differentiated	15	10	0.175	22	3	0.873
	Moderate differentiated	12	16		25	3	
	Poor differentiated	0	2		2	0	
**Mitosis ***	0-1/hpf	6	11	0.037**	15	2	0.826
	2-3/hpf	7	11		17	1	
	4-5/hpf	8	6		12	2	
	>5/hpf	6	0		5	1	
**Lymphoplasmacytic infiltration**	Weak	4	12	0.046**	16	0	0.230
	Moderate	12	11		19	4	
	Marked	11	5		14	2	
**Necrosis**	Yes	10	10	0.920	18	2	0.871
	No	17	18		31	4	
**Pattern of invasion ***	I	8	10	0.661	16	2	0.628
	II	12	8		18	2	
	III	6	8		13	1	

	IV	1	2		2	1	
**Stage of invasion** *	I	0	0	0.248	0	0	0.922
	II	14	20		30	4	
	III	12	8		18	2	
	IV	1	0		1	0	
**Treatment** *	Surgery	5	6	0.988	11	0	0.319
	Chemotherapy	2	2		3	1	
	Palliative treatment/support	16	18		29	5	
**Tumour stage**	I + II	8	8	0.602	15	1	0.488
	III + IV	13	18		27	4	

* Pattern of invasion also evaluated using the categorization of I + II vs III + IV (P=0.436 and P=0.893 for extent and intensity scores respectively) and I + II + III vs IV (P=0.634 and P=0.054); stage of invasion also evaluated using the categorization of I + II vs III + IV (P=0.139 and P=0.797); and treatment using the categorization of treatment in Surgery/ Chemotherapy vs Palliative treatment/support (P=0.980 and P=0.434). ** Pairwise multiple comparisons with bonferroni adjustment showed differences between number of mitosis between >5 vs 0-1 (P=0.042), squamous differentiation between 5-20% vs 0-5% category (P=0.034), and for lymphoplasmacytic infiltration between strong vs weak categories (P=0.043).

Suplemmental Table S5. Clinicopathological characteristics and Ki-67 expression comparison

		**Ki-67 Extent Score**			**Ki-67 Intensity Score**		
**Variables**		**≤49%**	**≥50%**	***P*-Value**	**(0/Weak/Moderate)**	**(Strong)**	***P*-Value**
**Gender**	Female	21	6	0.646	16	11	0.453
	Male	21	8		20	9	
**Age (years)**	<7	20	9	0.284	19	10	0.843
	≥7	22	5		17	10	
**Breed**	Small	9	0	0.136	7	2	0.385
	Medium	4	1		4	1	
	Large	6	5		5	6	
	Undetermined	19	6		17	8	
**Tumour Location**	Mouth (NOS)	8	2	0.697	8	2	0.452
	Gingiva	16	4		13	7	
	Tongue	7	4		7	4	
	Oropharynx	5	3		3	5	
	Palate	6	1		5	2	
**Histological type**	Papillary SCC	8	0	0.08	6	2	0.498
	Conventional SCC	34	14		30	18	
**Bone Invasion**	Yes	7	3	0.690	8	2	0.257
	No	35	11		28	18	
**Vascular Invasion**	Yes	3	1	1.000	4	0	0.125
	No	39	13		32	20	
**Histological grade (Anneroth)**	Well differentiated	15	3	0.326	9	9	0.128
	Moderate differentiated	27	11		27	11	
	Poor differentiated	-	-		-	-	
**Histological grade (Bryne)**	Well differentiated	19	5	0.632	13	11	0.278
	Moderate differentiated	22	8		21	9	
	Poor differentiated	1	1		2	0	
**Mitosis** *	0-1/hpf	13	4	0.580	8	9	0.059
	2-3/hpf	12	6		16	2	

	4-5/hpf	13	2		9	6	
	>5/hpf	4	2		3	3	
**Lymphoplasmacytic infiltration**	Weak	8	0	0.213	3	5	0.133
	Moderate	16	7		14	9	
	Marked	18	7		19	6	
**Necrosis**	Yes	14	5	0.872	13	6	0.646
	No	28	9		23	14	
**Pattern of invasion ***	I	18	0	0.007 **	10	8	0.517
	II	14	7		14	7	
	III	7	7		9	5	
	IV	3	0		3	0	
**Stage of invasion ***	I	0	0	0.148	0	0	0.693
	II	29	6		23	12	
	III	12	8		12	8	
	IV	1	0		1	0	
**Treatment ***	Surgery	9	2	0.092	7	4	0.725
	Chemotherapy	1	3		3	1	
	Palliative treatment/support	25	9		19	15	
**Tumour stage**	I + II	13	3	0.332	13	3	0.116
	III + IV	21	10		18	13	

* Pattern of invasion also evaluated using the categorization of I + II vs III + IV (P=0.067 and P=0.520) for extent and intensity scores respectively) and I + II + III vs IV (P=0.003 and P=0.352); stage of invasion also evaluated using the categorization of I + II vs III + IV (P=0.082 and P=0.775); and treatment using the categorization of treatment in Surgery/ Chemotherapy vs Palliative treatment/support (P=0.628 and P= 0.484). ** Pairwise multiple comparisons with bonferroni adjustment showed differences between pattern of invasion evaluated using the categorization of I vs III (P=0.008).

Suplemmental Table S6. Univariate analysis of cancer-specific survival (CSS) of clinical and histopathological variables.

Factors	Factors	N	Dead	CSS 1-Year *	CSS 2-Years *	CSS Mean CI 95% **	*P*-Value
**Gender**	Female	22	12	43.4	32.5	22.60±6.49 (9.88-35.33)	0.202
	Male	28	21	21.1	15.8	6.59±1.73 (3.19-9.98)	
**Age (years)**	<7	25	16	28.8	23.1	16.31±5.3 (5.84-26.78)	0.890
	≥7	25	17	31.9	21.2	8.85±2.11 (4.72-12.97)	
**Breed †**	Small	8	4	34.3	34.3	9.72±3.78 (2.30-17.14)	0.247
	Medium	3	1	66.7	66.7	11.32±3.81 (3.83-18.81)	
	Large	10	8	20.0	20.0	7.70±4.16 (0.00-15.86)	
	UB	22	14	28.7	28.7	14.73±5.73 (3.50-25.95)	
**Tumour Location**	Mouth (NOS)	7	4	33.3	33.3	9.01±3.17 (2.79-15.23)	0.643
	Gingiva	18	9	40.0	40.0	24.98±7.34 (10.58-39.38)	
	Tongue	10	8	25.0	0.00	6.26±2.69 (0.98-11.52)	
	Oropharynx	8	7	0.00	0.00	4.02±1.09 (1.89-6.16)	
	Palate	7	5	28.6	28.6	8.39±3.98 (0.60-16.18)	
**Histological type**	Papillary SCC	6	1	80.0	80.0	48.30±9.30 (30.07-66.53)	0.013
	Conventional SCC	44	32	24.1	13.4	6.94±1.43 (4.13-9.75)	
**Bone Invasion**	Yes	8	4	35.0	35.0	21.35±11.33 (0.00-43.56)	0.856
	No	42	29	30.4	20.2	10.45±2.20 (6.14-14.77)	
**Vascular Invasion**	Yes	4	2	50.0	50.0	11.71±5.29 (1.34-22.09)	0.689
	No	46	31	29.6	19.8	15.08±3.97 (7.30-22.87)	
**Histological grade**	Well differentiated	16	10	33.9	33.9	21.55±6.89 (8.04-35.06)	0.543

**(Anneroth)**	Moderate differentiated	34	23	28.7	16.4	7.97±1.75 (4.55-11.40)	
	Poor differentiated	0	0	0	0	0	
**Histological grade (Bryne)**	Well differentiated	22	16	55.8	16.7	12.20±4.88 (2.63-21.77)	0.112
	Moderate differentiated	26	15	44.7	26.1	10.95±2.21 (6.62-15.28)	
	Poor differentiated	2	2	0.00	0.00	1.35±0.15 (1.06-1.64)	
**Mitosis**	0-1/hpf	16	11	31.3	31.3	20.19±6.51 (7.43-32.94)	0.934
	2-3/hpf	15	10	23.5	0.00	6.58±2.28 (2.10-11.05)	
	4-5/hpf	13	7	46.0	34.5	13.85±4.75 (4.54-23.16)	
	>5/hpf	6	5	16.7	16.7	6.88±3.41 (8.53-23.65)	
**Lymphoplasmacytic infiltration**	Weak	15	13	31.0	31.0	6.18±1.87 (2.51-10.15)	0.448
	Moderate	21	11	40.3	32.3	21.56±6.66 (8.51-34.61)	
	Marked	14	9	20.0	10.0	6.33±1.95 (2.51-9.85)	
**Necrosis**	Yes	18	11	26.6	26.6	18.05±6.64 (5.05-31.06)	0.547
	No	32	22	32.0	18.3	9.94±2.53 (4.98-14.90)	
**Pattern of invasion**	I	15	5	61.9	61.9	37.74±7.40 (23.24-52.25)	0.011
	II	19	16	13.0	0.00	4.79±1.50 (1.84-7.74)	
	III	13	10	20.0	0.00	4.15±1.44 (1.33-6.97)	
	IV	3	2	33.3	33.3	7.84±5.90 (0.00-19.41)	
**Stage of invasion**	I	0	0	0	0	0	0.009
	II	31	17	42.9	25.0	19.58±5.77 (8.27-30.88)	
	III	18	15	12.3	12.3	4.75±1.90 (1.01-8.48)	
	IV	1	1	0.00	0.00	76.0±0.00 (76.00-76.00)	
**Treatment**	Surgery	11	4	55.6	55.6	20.74±4.95 (11.03-30.44)	0.048
	Chemotherapy	4	4	0.00	0.00	3.46±1.60 (0.33-6.58)	
	Palliative treatment/support	35	25	26.9	14.4	11.97±4.12 (3.89-20.05)	
**Tumour stage †**	I	6	1	100	66.7	45.47±10.80 (24.29-66.64)	0.001
	II	9	4	50.8	50.8	12.73±3.45 (5.97-19.48)	
	III	12	8	13.9	13.9	4.50±1.41 (1.73-7.26)	
	IV	17	16	0.00	0.00	2.23±0.53 (1.20-3.27)	
**Tumour stage †**	I + II	15	5	64.6	48.5	32.53±8.61 (15.66-49.40)	0.001
	III + IV	29	24	99.0	99.0	3.45±0.74 (2.00-4.89)	

Legend: UB, undetermined breed (including mixed breeds); NOS, not otherwise specified; SSC, squamous cell carcinoma; hpf, high power field. * Cumulative proportion (%) of survival time; ** mean for survival time in months (cancer-specific survival); † Information not available for some patients.
